# Tristate Meetings at Kansas City

**Published:** 1916-09

**Authors:** C. N. Johnson


### THE TRISTATE MEETINGS AT KANSAS CITY.

On March 20-26, 1916, the three states of Kansas, Oklahoma, and Missouri joined in a meeting at Kansas City, Mo., which will be memorable in the history of dentistry in the West. More than one thousand men representing eighteen states were in attendance, and there was an intensity of interest throughout the week that surpassed anything ever witnessed at a similar gathering of dentists. From Monday when the meeting opened till Saturday afternoon when it closed there was never a lapse in the attendance nor a lag in the interest. The audience would sit for three hours in succession listening to lectures and then ask for more. The meeting was conducted on the lecture system entirely, no clinic being given. The lecturers were Drs. Thomas P. Hinman, Atlanta, President of the National Dental Association; Forrest H. Orton, St. Paul; M. L. Rhein, New York; Richard H. Riethmuller, Philadelphia; Weston A. Price, Cleveland; and J. P. Buckley and C. N. Johnson, Chicago.

To go into details regarding the meeting would be superfluous and in fact impossible, on account of the many and varied features of interest; but to show something of the spirit manifest it is only necessary to state that after Dr. Price's lectures more than $6,000.00 was raised for the Research Fund of the National Dental Association. When it is realized that each man at the meeting had paid $5.00 for the privilege of attending the lectures, such a contribution as this speaks volumes for the liberality of the dentists of the West.

A conspicuous feature of the meeting was the excellence of the reports in the daily press of Kansas City. Throughout the week the reports were interesting, dignified and instructive; and the press reporters were apparently bent on giving only accurate and faithful information as to the matters emphasized by the speakers, instead of straining at effects through the medium of buffoonery, as has too frequently been the case in the past. Hats off to the Kansas City papers.

The success of this Tri-State meeting was due to the untiring efforts of the officers and committees in charge. They all worked together with a will and each is entitled to a full share of praise. If any one man could be singled out of the whole noble band of workers for special commendation, it would be Dr. C. R. Lawrence of Enid, Oklahoma—the Secretary-Treasurer of the Organization Committee. Dr. Lawrence worked in season and out, not only with earnestness, but with a particular genius for such work—to the end that at the banquet he was presented with a beautiful gold watch by his confreres as a token of their esteem.

Altogether this meeting will long stand out as an important mile stone in dental progress, and our congratulations are herewith extended to those associated in its management. The proceedings are to be published in our esteemed contemporary the *Western Dental Journal* of Kansas City, whose editor is Dr. Charles Channing Allen, Chairman of the Organization Committee.—Dr. C. N. Johnson, in August *Review*.

